# Mechanistic Aspects and Therapeutic Potential of Quercetin against COVID-19-Associated Acute Kidney Injury

**DOI:** 10.3390/molecules25235772

**Published:** 2020-12-07

**Authors:** Lúcio Ricardo Leite Diniz, Marilia Trindade de Santana Souza, Allana Brunna Sucupira Duarte, Damião Pergentino de Sousa

**Affiliations:** 1 Department of Nursing, College of Nordeste da Bahia, 48590-000 Coronel João Sá, Bahia, Brazil; 2 Pharmacy Center, Uninassau University, 49075479 Aracaju, SE, Brazil; biomari@hotmail.com; 3 Department of Pharmaceutical Sciences, Federal University of Paraíba, 58051-970 João Pessoa, PB, Brazil; allanabrunna@gmail.com (A.B.S.D.); damiao_desousa@yahoo.com.br (D.P.d.S.)

**Keywords:** natural products, medicinal plants, flavonoids, antioxidant, anti-inflammatory activity, nephroprotective activity, renoprotective activity, SARS-CoV, Middle East respiratory syndrome virus, coronavirus

## Abstract

The inflammatory mediator and oxidant agent storm caused by the SARS-CoV-2 infection has been strongly associated with the failure of vital organs observed in critically ill patients with coronavirus disease 2019 (COVID-19) and the death of thousands of infected people around the world. Acute kidney injury (AKI) is a common renal disorder characterized by a sudden and sustained decrease in renal function with a critical influence on poor prognosis and lethal clinical outcomes of various etiologies, including some viral infection diseases. It is known that oxidative stress and inflammation play key roles in the pathogenesis and development of AKI. Quercetin is a natural substance that has multiple pharmacological properties, such as anti-inflammatory action, and is used as a dietary supplement. There is evidence of the anti-coronavirus activities of this compound, including against the target SARS-CoV-2 3CLpro. The ability to inhibit coronavirus and its inflammatory processes is strongly desired in a new drug for the treatment of COVID-19. Therefore, in this review, the dual effect of quercetin is discussed from a mechanistic perspective in relation to AKI kidney injury and its nephroprotective potential to SARS-CoV-2 patients.

## 1. Introduction

Coronavirus disease 2019 (COVID-19) has killed more than one million people with SARS-CoV-2 worldwide, and it continues creating great concern for the world medical community due to the continually elevated number of deaths caused by COVID-19 [1,2,3]. Recent clinical reports have indicated that COVID-19 may lead to severe and fatal respiratory complications and even to other organ failures, such as renal failure [4,5,6]. The increasing number of studies that relate a substantial number of patients with SARS-CoV-2 with acute kidney injury (AKI) have suggested that the impairment of renal functions, such as the retention of waste products of cellular metabolism and the lack of maintaining fluids, electrolytes, and acid–base homeostasis, contributes to the worsening of clinical outcomes and consequently to the lethality of COVID-19 [7,8,9,10]. AKI is a common renal disorder characterized by a sudden and sustained decrease in renal function with a critical influence on poor prognosis and lethal clinical outcomes of various etiologies, including cardiovascular diseases, diabetes, and sepsis [11,12].

The unavailability of specific treatment and the inability to accurately predict a timeline for an effective vaccine against COVID-19 [13,14,15] make it extremely necessary to test compounds with therapeutic properties whose pharmacological safety and efficacy have been previously tested to inhibit, or at least attenuate, organ disorders strongly associated with the worsening of clinical symptoms and lethality of COVID-19, including kidney disorders [8,9,10,16]. In this context, quercetin may be a very helpful therapeutic tool for the treatment of AKI induced by COVID-19. Quercetin is a widespread flavonoid found in a large variety of medicinal plants and dietary supplements present in oriental and occidental diets, such as green teas, oranges, lettuces, potatoes, onions, and tomatoes [17,18]. Furthermore, it has inhibitory activity against severe acute respiratory syndrome coronavirus (SARS-CoV) and Middle East respiratory syndrome coronavirus (MERS-CoV) [19]. Numerous studies have shown the nephroprotective effects of quercetin against obstructive, oxidative, and inflammatory renal damage in a large spectrum of experimental models of AKI [20,21,22,23,24]. The antioxidant and anti-inflammatory properties have been credited as the main mechanisms by which quercetin inhibits the renal damages [19,22,25,26,27,28,29]. 

The present study aims to give an overview of the safety and effectiveness of quercetin on AKI induced by very diverse nephrotoxic stimuli. Its therapeutic potential for COVID-19-related renal injury could be evaluated through an evaluation of the current literature pertaining to the various cellular and molecular targets of AKI.

## 2. Materials and Methods

The present article was prepared based on a survey of the literature pertaining to quercetin and AKI. The search, performed in the PubMed database, included studies published until August 2020 and used the following keywords: quercetin, coronavirus, COVID-19, SARS-COV-2, AKI, acute renal failure, virus-induced AKI, SARS-COV-2-induced AKI, and COVID-19-induced AKI. Only studies in which the renoprotective effects of quercetin were investigated under experimental or clinical AKI in accordance with Kidney Disease: Improving Global Outcomes (KDIGO) stages 1, 2, or 3 definitions were selected. Results obtained from quercetin-derived compounds or a combination of quercetin with other bioactive drugs on nephroprotective properties were not considered. The scientific publications were selected from studies published in the English language.

## 3. General Aspects of Acute Kidney Injury

AKI is a common renal disorder characterized by a sudden (1 to 7 days) and sustained (more than 24 h) decrease in renal function with a critical influence on poor prognosis and lethal clinical outcomes of various etiologies and that contributes to the risk of long-term morbidity and death [30,31,32,33]. AKI is directly associated with serious clinical complications, including the retention of waste products of cellular metabolism that leads to the rise in cytotoxic substance levels, the alteration of drugs’ concentrations that reduces their pharmacological safety, and the lack of maintaining fluids, electrolytes, and acid–base homeostasis, which in turn are closely linked to alterations in blood pressure control, neuromuscular excitability, and metabolic disturbance [6,7,9]. In addition, AKI produces diverse inflammatory mediators and vasoactive agents that act on the kidney as well as distant organs, playing an important pathological role in the genesis and development of several diseases [34,35]. The course of AKI can vary from single hit and complete recovery to multiple hits that might lead to renal failure requiring dialysis, the development and progression of chronic kidney disease (CKD), and death [32,36]. Around the world, the incidence of AKI is about 20% of hospitalized patients and can reach up to more than 50% of all critically ill patients in intensive care units (ICUs) [33,37,38]. The development of AKI is directly associated with the prevalence and clinical outcome of hypertension, diabetes, and sepsis in ICU settings [32,39]. In a study carried out by Dylewska et al. [40], it was observed that the prevalence of hypertension was 70%, with the highest rate in post-renal AKI (85%), followed by renal AKI (75%), and pre-renal AKI (30%). In recent years, the world incidence of diabetes-induced AKI of hospitalized patients has reached more than 30% in diabetic adults, accompanied by an overall increased morbidity [35]. Regarding sepsis, up to 57% of all critically ill patients suffer from AKI [41,42]. Moreover, sepsis appears to be the most frequent contributing factor for the development of AKI. Studies indicate that AKI is caused by about 50% of sepsis cases worldwide [33,39]. 

Preliminary findings have shown that the etiology and prevalence of AKI vary, mainly due to different aspects of the sample evaluated (i.e., animal species, sex, age, and comorbidity), differences in the income levels of countries (with low-to-middle income countries showing more severity and a higher incidence of AKI than high-income countries), and differences in AKI classification criteria [32,38,43,44]. Throughout the last two decades, the AKI definition has been mainly based on the RIFLE (Risk, Injury, Failure, Loss, End-Stage Kidney Disease), Acute Kidney Injury Network (AKIN), and Kidney Disease: Improving Global Outcomes (KDIGO) criteria and stages [43,44]. Until 2004, the diagnosis of AKI was based on urine output as well as blood urea nitrogen (BUN) and serum creatinine (SCr) levels. The lack of a precise biochemical definition for AKI led the Acute Dialysis Quality Initiative group to create the RIFLE criteria in 2004, establishing a multidimensional staged definition. RIFLE uses glomerular filtration rate (GFR) criteria in addition to SCr and urine output criteria. The Risk, Injury, and Failure stages were determined by increases in SCr ≥ 1.5-, 2-, and 3-fold from a known baseline, respectively [43,44,45]. In 2007, the Acute Kidney Injury Network (AKIN) definitions were created with criteria driven by observations of minor increases in SCr over a shorter time period (48 h). The Risk, Injury, and Failure categories of the RIFLE definition were replaced by stages 1, 2, and 3 and the GFR criteria were abandoned in the AKIN definitions. The diagnosis of AKI was assessed by a rise in SCr ≥ 0.3-mg/dl over 48h in AKIN stage 1 and an absolute rise in SCr > 26.4 μmol/l was added to the relative increase of 100% and 200% in SCr compared to baseline in AKIN stages 2 and 3, respectively. The current definition created by KDIGO is similar to the AKIN definition but the time frame is extended from 48 h to 7 days. The decrease in urinary output to less than 0.5 mL kg^−1^ h^−1^ for 6 h was also similar to the RIFLE and AKIN definitions [30,43,44,45,46]. The actual criteria used to define AKI and its stages are not entirely accepted by all researchers and clinicians, who consider SCr and urine output more accurate markers of kidney dysfunction than AKI; consequently, both parameters are not ideal markers of AKI [47]. Over the last few years, many urine or blood biomarkers of AKI have been proposed as adjunct diagnostics to SCr and urinary output to improve the early detection, differential diagnosis, and prognostic assessment of AKI [48,49]. The following are among the novel biomarkers of AKI:

Neutrophil gelatinase-associated lipocalin (NGAL): lesions in the thick ascending loop of Henle and the intercalated cells of the collecting duct [49,50,51]. 

Kidney injury molecule-1 (KIM-1): lesions in proximal tubule cells [44,49,52]. 

Interleukin-18 (IL-18): lesions in collecting duct and tubular epithelial cells [48,49].

Liver-type fatty acid-binding protein (L-FABP): lesions in proximal tubule cells [49].

Calprotectin (S100A8/9): lesions in collecting ducts and filtrating immune cells [48,50].

In general, the mechanisms involved in the pathophysiology of AKI are attributed to renal damage caused by ischemic process, oxidative stress, and inflammation [30,53]. Many pathophysiological mechanisms contribute to kidney tubular and endothelial cell injury and consecutive renal dysfunction observed in ischemic AKI, including reduced renal blood flow (RBF), renal tubular apoptosis, necrosis, and inflammation [54,55]. In RBF-independent microcirculatory dysfunction, inflammatory mediators, immune cell infiltration, and the deregulation of nitric oxide synthase lead to the redistribution of blood flow from renal medulla to the cortex, the deterioration of microcirculatory oxygenation, tubular cell damages, and the formation of reactive oxygen species (ROS) and reactive nitrogen species (RNS) [35,41,54].

## 4. Viral Infections and Acute Kidney Injury

Kidneys are high-risk organs for viral invasion, and the presence of AKI represents poor prognosis and potentially fatal outcome in several virus diseases [56]. Therefore, avoiding or even controlling the progression of AKI during viral infection have become challenges for the medical community. Various kidney diseases are associated with viral infections, including infection caused by the HIV virus [57], Epstein–Barr virus [58], parvovirus B19 [59], hepatitis B virus (HBV) [60], hepatitis C virus (HCV) [61], Zika virus [62], dengue virus [63], and influenza A (H1N1) virus [64]. A large spectrum of prevalence and clinical manifestations are observed among viral diseases, ranging from mild and rare AKI caused by acute symptomatic Epstein–Barr virus infectious mononucleosis [58] to rapid deterioration of renal function, often resulting in the requirement of renal replacement therapy, as observed in hantavirus-type infection [51,58,65,66]. 

Virus-associated glomerular disease, such as membranous glomerulopathy, glomerulosclerosis, and membranoproliferative glomerulonephritis associated with HCV infection [67,68,69], as well as interstitial nephritis and necrotizing tubulointerstitial nephritis are common clinical manifestations caused by BK virus, cytomegalovirus, and adenovirus infections [56,59]. AKI-associated nephritis is also a common renal disorder observed in patients with hepatitis B virus (HBV)-related acute-on-chronic liver failure (ACLF) and adenovirus infections [60,70,71]. In the last decade, high incidences of AKI have been found in critically ill patients with severe acute respiratory syndrome (SARS) and influenza A (H1N1), while in turn a strong relation between AKI and mortality rate was also observed [72,73].

The pathological mechanisms behind virus-associated kidney injury manifestations are either immune complex deposition or immune reactions resulting in glomerular membrane proliferation, based on severe coagulopathy, endothelial damage, and increased vascular permeability [70,74,75]. Massive releases of myoglobin-, cytokine-, and humoral factor-mediated acute interstitial nephritis play an important role in many viral infections [67,76]. Histological investigation shows the presence of acute distal tubular necrosis and viral particles in epithelial cells as well as the Bowman’s capsule found in many viral diseases, which differ significantly from those reported in bacterial infection characterized by increased rates of apoptosis in renal tubular epithelia as well as leucocytic infiltration in glomeruli and capillaries [56,68,70].

One relevant aspect in the incidence and outcome of AKI in viral infection is the presence of comorbidities and coinfection, particularly in those patients with a longer ICU stay and/or immunodeficiency [77,78]. In this context, HIV-infected patients may be predisposed to acquiring new viral coinfections added to HIV-associated nephropathy (HIVAN), immune complex kidney disease, thrombotic microangiopathy, and drug-related injury [75]. Thus, the kidney diseases found in HIV patients, such as immunotactoid glomerulopathy and fibrillary glomerulonephritis, are usually more severe and complex than other virus-associated kidney injuries [79].

An important feature of virus-associated kidney injury is the presence of virus in the urine for long periods after initial infection [80]. Considering that neither CD4+ nor CD8+ T cells are detected in the kidney, even after a long period of viral infection, increasing evidence that virus can infect renal tubules without the recruitment of immune cells as a viral strategy for persistent survival has been reported [54,75,79]. Ou et al. [80] investigated the replication strategies and immune responses of kidney caused by the duck hepatitis A virus (DHAV) up to 280 days after virus infection. In accordance with preliminary findings that cytokines, interferons, and interleukins are vital for both antiviral responses and the pathogenesis of viral infection, the results showed a strong cytokine storm, including type I (IFN-α/β) and type II (IFN-γ) IFNs, Th1-related ILs (IL-1β/2/6), and Th2-related ILs (IL-4), followed by a sudden decrease in virus loads in the mesangial cells and vascular endothelial cells for early infection [80]. The cytokine storm was consistent with both viral decrease and kidney injury, which combined with the histopathological changes indicated a double-edged sword for the host defense, since it not only enhanced viral clearance but also had a pathogenic effect [54,56].

## 5. Coronavirus and Acute Kidney Injury

Even though a better understanding of the pathophysiologic mechanisms of COVID-19-associated AKI is needed, there is increasing evidence that AKI is prevalent in critically ill patients with SARS-CoV-2 infection and it is closely associated with the severity of COVID-19 [11,12,81,82]. Moreover, clinical data have suggested that AKI represents poor prognosis and is associated with high mortality of patients with SARS-CoV-2 in ICU settings, especially in those with underlying comorbidities and requiring renal replacement therapy [3,9]. For example, a study that analyzed the clinical characteristics and complications in fatal cases with coronavirus disease 2019 (COVID-19) in Renmin Hospital of Wuhan University in China reported that 14 patients suffered from renal injury after the infection of SARS-CoV-2 in 92 deceased patients with COVID-19. AKI was determined based on levels of SCr, BUN, and GFR found in these 14 patients and compared with the cases without renal injury. The median levels of SCr, BUN, and GFR in cases with renal insufficiency were 262 μmol/L, 30 mmol/L, and 18 mL/min, respectively [83]. In a study performed with 138 patients with SARS-CoV-2 hospitalized in the ICU of Mount Sinai Hospital (New York, NY, USA), 49 cases of AKI, characterized by an increase in the SCr level from approximately 1.0 to 8.0 mg/dL, a rise in the BUN level from about 20 to 100 mg/dL, and an increase in the serum phosphorus level > 8 mg/dL, over three days, were diagnosed among patients with COVID-19 by the Nephrology Department of the hospital. The magnitude of the variance of renal function parameters depends on certain factors, such as age and pre-existing comorbidities. Based on the serum phosphorus level > 10 mg/dL and a parameter arbitrarily chosen, 9 of 49 patients were found with AKI, with a mean AKI duration before phosphorus levels reaching 10 mg/dL after six days. Among the 9 patients, 6 died, 2 recovered, and 1 experienced no change [16].

In an attempt to obtain a definite conclusion on the association between AKI and the risk of mortality in patients with COVID-19, Yang et al. [83] performed a systematic review and meta-analysis study, in which a total of 24 studies published up to 26 April 2020 were evaluated, by searching the PubMed, Web of Science, and China National Knowledge Infrastructure databases, involving 4963 confirmed COVID-19 patients. The results showed that the incidence of AKI was 1.3%, 2.8%, and 36.4% in mild or moderate cases, severe cases, and critical cases, respectively. Meanwhile, the incidence of AKI was 52.9% and 0.7% in non-survivors and survivors, respectively [84]. 

In a recent study based on the analysis of several clinical reports, Gagliardi et al. [85] related that up to 15% of the hospitalized COVID-19 patients had at least one kidney abnormality represented by increased BUN and reduced GFR, as well as 26–63% of patients presented proteinuria at admission or developed proteinuria during their stay in hospital. Moreover, the incidence of AKI in COVID-19 patients varied from 0.5% to 23%, with an interval from baseline visit to the onset of AKI of 7–15 days in median, and that mortality from the COVID-19 patients who developed AKI could be up to 13 times higher than those infected patients without clinical signs of AKI. In addition, the authors suggested that the high prevalence of kidney involvement at hospital admission of some COVID-19 patients may be associated with some factors, including report of previous renal impairments, patients’ age, severity of illness, and presence of diabetes and/or heart failure, which are all risk factors for AKI that contribute to a pro-inflammatory state with functional defects in their immune system, worsening the clinical conditions of COVID-19 patients [85].

It should be noted that, in spite of strong evidence of increasing kidney dysfunction caused by COVID-19 and of AKI being closely associated with the severity and prognosis of COVID-19 patients, the actual AKI incidence, in particular in ICUs, remains uncertain and may have been underestimated due to different causes, including the design of the studies, the lack of clear operational AKI definitions, the reported AKI stages, and the timeline of AKI onset incidences [4,5,8,81,82].

## 6. Quercetin: A Natural Polyphenol Compound with Pharmacological Properties for the Treatment of COVID-19-Induced Morbidities

Quercetin is a widespread flavonoid, more specifically a flavonol (3,5,7,3′,4′-pentahydroxyflavone), with a broad range of pharmacological properties and is found in a large variety of medicinal plants and dietary supplements [17,18]. Very diverse foods containing quercetin are present in oriental and occidental diets. Among food-based sources of quercetin are black and green teas, nuts, apples, grapes, berries, oranges, lettuces, potatoes, onions, and tomatoes. Quercetin is also available at the market and can be used in the isolated form [17,18,86,87]. 

Preliminary investigations on the pharmacokinetic properties of oral and intravenous quercetin, at dose levels varying from 8 to 2000 mg/m^2^, in humans have indicated that quercetin has a very poor oral bioavailability (~2%), with low oral absorption, ranging from 3% to 17%, distribution and volume of distribution of 0.7–7.8 min and 3.7 L/m^2^, respectively, and extensive metabolism and/or rapid elimination, as indicated by clearance of 0.23–0.84 L/min/m^2^ and elimination half-life of 3.8–86 min [86,87,88,89,90]. In addition, the reported Cmax and Tmax of quercetin are 2.3 ± 1.5 µg/mL and 0.7 ± 0.3 h, respectively [89]. The low bioavailability of quercetin has limited its oral use in experimental and pharmaceutical studies. Consequently, parenteral routes of administration have been commonly used to investigate the biological properties of quercetin [86,87]. However, conjugated forms of quercetin’s glycosides (with different sugar types and sugar group conjugation sites or pharmaceutical formulations in which it is associated with materials that enhance the solubility and/or dissolution rate of lipophilic drugs in aqueous media such as cyclodextrin) have shown improved bioavailability and are frequently used to evaluate pharmacological activities of oral quercetin [88,89]. Previous studies have indicated that quercetin’s safety dose is about 1000 mg/m^2^ and its main toxicological effects are emesis, hypertension, and hypokalemia [90]. 

In recent years, diverse pharmacological properties have been attributed to quercetin, such as cardioprotection in spontaneous and experimentally induced hypertension, inhibition of secondary biliary cirrhosis, prevention of platelet aggregation, as well as antiangiogenic, anticancer, antiallergic, antiulcer and anti-inflammatory properties [17,18,86,91,92,93,94]. In general, the majority of the beneficial effect of quercetin is associated with antioxidant effects mainly through free radical scavenging and metal chelation and anti-inflammatory properties through NF-κB inhibition [27,95,96,97].

In this context, quercetin has been extensively studied for essential pharmacological properties requested in the treatment of Covid-19 and coronavirus-induced organ injury, such as antiviral, anti-oxidative and anti-inflammatory activities [13,15,94]. Previous studies have reported that quercetin and quercetin-derived compounds, such as quercetin-3-β-galactoside, display potent inhibitory effect on 3C-like protease (3CL(pro)) of SARS-CoV and MERS-CoV, a vital enzyme for viral replication, leading to inhibition of both coronavirus replications [98,99].

Multiple studies in different cell types and in both animal and human models have supported the long-lasting anti-inflammatory properties of quercetin [17,18,94]. Briefly, in vitro studies have shown that quercetin inhibited both cyclooxygenase and lipoxygenase activities in guinea pig epithelial cells [100], reduced mRNA levels and TNFα, IL-1α, and apoptotic neuronal cell death induced by microglial activation [100] as well as inhibiting TNF-α and IL-8 production induced by lipopolysaccharide (LPS) in macrophages and lung A549 cells, respectively [101]. In rat liver epithelial cells, quercetin promoted the inhibition of arsenite-induced COX-2 expression mainly by blocking the activation of the PI3K signaling pathway [102]. It also inhibited Src- and Syk-mediated PI3K-(p85) tyrosine phosphorylation and subsequent TLR4/MyD88/PI3K complex formation that limits the activation of downstream signaling pathways in RAW 264.7 cells [103]. Quercetin inhibited the IL-1-induced IL-6 secretion in mast cells [104]. Other anti-inflammatory mechanisms, such as downregulation of MMP-1, VCAM-1, and CD80 expression produced by quercetin, were observed in human umbilical skin and vein endothelial cells [101]. Various animal experiments have supported the in vitro anti-inflammatory properties of quercetin. Studies using rodents showed a modulation of prostanoid synthesis and cytokine production promoted by quercetin. Stewart et al. [104] showed that quercetin caused a decrease in interferon-γ, interleukin-1α, and interleukin-4 from C57BL/6J mice, corroborating with the inhibition of macrophage-derived cytokines and nitric oxide produced by quercetin in Lewis rats previously reported [95]. In humans, the anti-inflammatory effect of quercetin remains not fully consistent with results from cell (in vitro) and animal (in vivo) studies [87]. According to data of studies with humans, the anti-inflammatory properties of quercetin appear to be dependent on the type of subject and their level of health. For example, it was observed that there was no significant anti-inflammatory effect promoted by quercetin in cyclists after intense exercise [105], whereas quercetin treatment promoted anti-inflammatory effects, evidenced by a decrease in nitric oxide, C-reactive protein, and γ-glutamyl transferase activity in elderly human subjects [106]. Thus, the outcomes in humans need to be carefully evaluated and further investigation must be done in order to support the results of tests in cells and animals aiming at a broad application in the future.

Polyphenolic compounds, such as the flavonoids, commonly show antioxidant properties due to the presence of phenolic rings that promote the electron donation and hydrogen atom transfer to free radicals, acting as free-radical scavengers, reducing agents, and quenchers of single oxygen formation [93,107]. The antioxidant properties of quercetin have been strongly evidenced in studies using cells and animals. Quercetin (10 μmol/L) inhibited oxidative stress promoted by H_2_O_2_ in HepG2 cells [108,109]. Chen et al. [109] reported that quercetin decreased apoptosis and ROS production and increased SOD levels in intestinal porcine enterocyte cells [109]. In addition, Meng et al. [110] showed that quercetin produced an increase in SOD, CAT, and glutathione peroxidase (GSH-Px) levels accompanied by a decrease in lipid peroxidation in rats subjected to experimental sepsis and chronic prostatitis/chronic pelvic pain syndrome [111].

In addition to the antiviral, anti-inflammatory and antioxidant actions of quercetin, some mechanisms of quercetin’s action on the regulation of ion transporters and channels are important for understanding vasodilation and the increase in blood flow involved in protective properties of quercetin against organ injury [112]. It has been demonstrated that quercetin activates Na^+^-K^+^-2Cl^−^ cotransporter 1 (NKCC1) that leads to elevation of the cytosolic Cl^−^ concentration ([Cl^−^]c), which in turn downregulates gene expression of ENaC and decreases Na^+^, K^+^-ATPase activity [113,114]. These actions contribute to the reduction in vascular contraction and the renal Na+ reabsorption that explain, at least in part, the effects of quercetin on the regulation of blood pressure and renal function [17,115].

## 7. Quercetin and Acute Kidney Injury

Over the last few years, the renoprotective effect of quercetin has been tested in a wide range of experimental models of AKI [20,46,53,116]. In general, the studies have shown that quercetin treatment, at doses varying from 1 to 100 mg/kg, promotes significant nephroprotective activity, evidenced by inhibition or attenuation of the increase in classical biomarkers of AKI in renal injuries of toxic, obstructive, and inflammatory origins [20,94,115,116]. As shown in Table 1, several studies using AKI models created for several nephrotoxic compounds, including cisplatin [24,117], methotrexate [118,119], contrast [120], NaF [121], HgCl2 [122], manganese [123], cadmium [124], and ciprofloxacin [29], showed that pretreatment with quercetin orally avoided an increase in BUN and SCr and a lower decrease in the GFR of animals that received a high dose of a nephrotoxic agent. Similarly, quercetin treatment improved renal function, evidenced by lessened renal pathologies, and lowered BUN and SCr levels, and histological integrity has been reported in an experimental model of renal ischemia and reperfusion (I/R) injury and sepsis-induced AKI, the most common causes of AKI in ICUs worldwide [21,53,115]. Gholampour and Sadidi [125] related that oral treatment with quercetin inhibited kidney dysfunction, characterized by a significant decrease in creatinine clearance and histological damages in the I/R-induced AKI, by clamping renal arteries for 45 min followed by 24 h reperfusion [125]. Previously, Shoskes [21] had reported the renoprotective properties of quercetin on ischemia/reperfusion in rats who underwent 30 min of left renal pedicle occlusion with simultaneous right nephrectomy [21]. 

The nephroprotective effects of quercetin appear to be directly associated with the reduction of oxidative stress [53,94]. The improvement of renal function promoted by quercetin treatment is accompanied by control of the oxidant–antioxidant balance [26,94]. Data from various studies with different nephrotoxic origins have shown that quercetin inhibits a decrease in glutathione peroxidase, superoxide dismutase, and catalase activities and an increase in malondialdehyde levels (lipid peroxidation) in AKI caused by toxins or drugs, ischemia/reperfusion, and sepsis [25,26,125,126,127].

Another very important feature of the mechanism underlying the renoprotective property of quercetin has been attributed to its actions on different targets of inflammatory response involved in the genesis and progression of renal injuries [53,87,92]. Pretreatment with quercetin significantly inhibited TNF-α, IL-1β, and IL-6 production in mice with LPS-induced AKI [128,129]. In addition, quercetin also significantly inhibited TLR4, MyD88, and TRAF-6 expressions and NF-κBp65 activation in the kidneys of rats with LPS challenge [23,128]. Shu et al. [130] showed that quercetin blocked CD38, possessing ADP-ribosyl cyclase (ADPR-cyclase) and cyclic ADP-ribose hydrolase (cADPR-hydrolase), in a mouse model with LPS-induced AKI [130]. In addition, quercetin treatment promoted a decrease in the renal levels of iNOS and IL-12 and the excessive accumulation of extracellular matrix and interstitial fibrosis by antagonizing NF-κB signaling activation and TGF-β1/Smad2/3 signaling [29]. It was also observed that quercetin increased AMPK phosphorylation, inhibited mTOR phosphorylation, and activated autophagy observed in the kidneys of I/R mice, suggesting that quercetin might protect the kidney against the ischemic process by activating the AMPK-regulated autophagy signaling pathway [131].

Data from animal models of AKI and human biopsies have shown that macrophage is a major contributor to the inflammatory response to AKI. There are growing sources of evidence that quercetin ameliorates kidney injury via modulating macrophage polarization, which appears to be associated with downregulated activities of NF-κB p65 and IRF5, as well as by inhibiting ASK1/JNK3/caspase-3 by enhancing the Akt signaling pathway [28,132,133]. Quercetin also inhibited the infiltration of CD68+ macrophages, the proportion of F4/80+/CD11b+/CD86+ macrophages, and the polarization of F4/80+/CD11b+/CD206+ M2 macrophages in cultured macrophages from kidneys and spleens in mice after LPS injection, indicating a quercetin-induced inhibitory effect on inflammatory macrophage polarization [100,134,135]. Recently, Tan et al. [24] showed that quercetin reduced inflammatory mediators in LPS-induced bone marrow-derived macrophages (BMDMs) and in a cisplatin-induced AKI model, which were accompanied by downregulation of protein levels of Mincle, phosphorylation of Syk and NF-κB in kidney macrophage I, and upregulation of M2 macrophage activity [24,28].

## 8. Quercetin: A Helpful Therapeutic Drug Against SARS-COV-2-Induced AKI?

Quercetin is a safe widespread flavonoid, found in many medicinal plants and dietary supplements, that presents a broad range of biological effects in cells and animals [100]. Over the last few years, numerous studies have shown a significant renoprotective effect of quercetin against kidney injury induced by different nephrotoxic agents (see Table 1). As outlined above, quercetin showed nephroprotective effects against kidney injury directly associated with oxidative stress and inflammatory response, which lead, respectively, to the rise in renal levels of ROS/RNS and the massive release of inflammatory mediators, in very diverse animal models of AKI [17,56,87]. Quercetin was effective in blocking or attenuating kidney lesions and dysfunctions caused by sepsis, diabetic nephropathy, ischemia/reperfusion as well as renal damage induced by varied nephrotoxic substances [26,27,29,115]. In general, the renoprotective effect of quercetin is associated with antioxidant effects mainly through free radical scavenging and metal chelation [93], and anti-inflammatory properties by modulating macrophage polarization, via downregulated activities of NF-κB p65 and IRF5 [23,24]. 

We can take into account the following facts: the frequent onset of AKI in patients with SARS-CoV-2 represents a life-threatening complication and increased risk of death [4,5,6,7,8,9]; the unavailability of a specific treatment for COVID-19 and its clinical complications [1,2,3]; and experimental evidence of the renoprotective properties of quercetin [23,24,25,26,27,28]. The underlying mechanisms by which quercetin promotes renoprotection act directly on important mediators of the pathophysiology of coronavirus-induced AKI [117,118,126,128,129] (see Section 4, Section 5, Section 6 and Section 7). Taken all together, it is plausible to consider quercetin as a promising drug to be tested against COVID-19-induced AKI in clinical trials. 

Even though the results suggest that quercetin exhibited renoprotection, anti-inflammation, and antioxidant in vitro (cells) and in vivo (animals) activities, it often happens that studies in humans do not totally support the results from tests in cells and animals [100]. Thus, some relevant factors related to both clinical trials and quercetin need to be further verified for a future broad application in humans. For example, the type of subject, their level of health, illness severity, and presence of comorbidities are some of factors that might affect the results and should be considered by physicians and researchers [85]. Moreover, route of administration, formulation, and toxicity are parameters linked to pharmacological properties of quercetin that must also be evaluated beforehand [86,87,88,89]. Many features of quercetin that may alter the extrapolation of experimental results to clinical trials have already been minimized, such as the association of quercetin with cyclodextrins to the formulation of the inclusion complexes that improve its aqueous solubility and dissolution rate, increasing the bioavailability and the oral use of quercetin [89]. The choice of therapeutic dose could be based on preliminary findings of quercetin’s safety dose of 945 mg/m^2^ and the effective dose of quercetin used in previous studies in humans [87,88]. 

**Figure 1:**
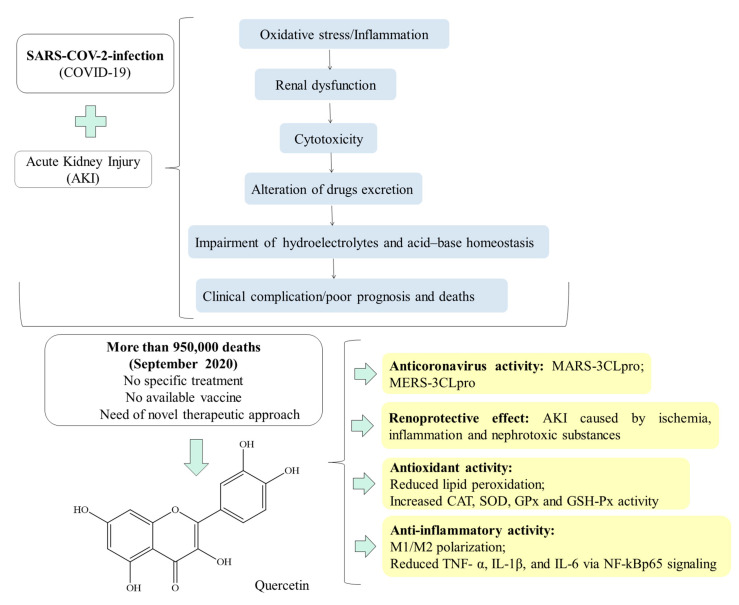
Quercetin as a helpful therapeutic drug against COVID-19-associated AKI.

In conclusion, the present review provides scientific evidence that supports the use of quercetin as a useful tool for the treatment of renal function impairment, to avoid the worsening of the clinical condition and, consequently, the short- and long-term morbidity and deaths of patients infected with SARS-COV-2 (see Figure 1). 

## Figures and Tables

**Table 1:** Pharmacological effect of quercetin in models of acute kidney injury (AKI).

Models of Acute Kidney Injury (AKI)	Pharmacological Effect	Mechanism	Reference
Ischemia/reperfusion-induced AKI models			
AKI induced by unilateral ischemia/reperfusion via left renal pedicle occlusion with simultaneous right nephrectomy	Renoprotective	Inhibited the decrease in creatinine clearance and tubular damage;	[21]
	Anti-inflammatory	Attenuated expression of normal T-cell expressed and secretion of monocyte chemoattractant protein-1, and allograft inflammatory factor.	
AKI induced by right renal and the left femoral blood vessels ischemia for 30 min followed by reperfusion in rats	Antioxidant	Reduced the renal cortex xanthine oxidase levels; Increased the renal cortex of superoxide dismutase, glutathione peroxidase, and catalase activities.	[116]
AKI induced by left renal pedicle occlusion for 45 min followed by 60 min of reperfusion with contralateral nephrectomy in rats	Anti-inflammatory	Reduced the TBARS, TNF-α levels, MPO activity and protein carbonyl;	[115]
	Antioxidant	Increased the glutathione levels and the superoxide dismutase and catalase activities.	
Renal pedicles occluded after nephrectomy; ischemia was given for 45 min followed by reperfusion for 24 h	Renoprotective	Attenuated the renal dysfunction via reduction in the SCr and BUN levels; Increased the creatinine and urea clearance; Reduced the morphological damage;	[135]
	Antioxidant	Reduced the elevated lipid peroxidation; Restored the depleted renal antioxidant enzymes, such as glutathione reductase.	
AKI induced by non-traumatic vascular clamp applied to the left renal pedicle for 2 h and allowed to reperfusion for 6 h	Antioxidant	Decreased the MDA levels; Increased the GSH levels;	[128]
	Anti-inflammatory	Decreased the number of apoptotic, p53-positive cells as well as reduced the NF-κB and eNOS expressions.	
AKI induced by clamping renal arteries for 45 min followed by 24 h reperfusion	Renoprotective	Sustained creatinine clearance and fractional excretion of sodium; Reduced tubular damage;	[125]
	Antioxidant	Increased glutathione peroxidase and catalase activities; Inhibited the decrease in malondialdehyde levels.	
Unilateral ureteral obstruction-induced renal fibrosis	Anti-inflammatory	Inhibited tubulointerstitial injury; Reduced the synthesis and release of inflammatory factors; Inhibited the infiltration of CD68+ macrophages in renal interstitium; Decreased levels of iNOS and IL-12, as well as the proportion of F4/80+/CD11b+/CD86+ macrophages; Inhibitory effects associated with downregulated activities of NF-κB p65 and IRF5, and thus led to the inactivation of upstream signaling TLR4/Myd88; Inhibited the polarization of F4/80+/CD11b+/CD206+ M2 macrophages; Reduced excessive accumulation of extracellular matrix and interstitial fibrosis by antagonizing the TGF-β1/Smad2/3 signaling.	[28]
**Drug- or Toxin-induced AKI models**			
Fe-NTA-Induced AKI	Renoprotective	Decreased the BUN and SCr; Preserved the normal renal morphology;	[126]
	Antioxidant	Reduced the lipid peroxidation; Restored the depleted renal antioxidant enzymes, such as glutathione reductase, catalase, superoxide dismutase, and glutathione.	
Cadmium-induced AKI	Renoprotective	Reduced of BUN, SCr and uric acid levels; Protected against the Cd-induced pathological condition, as tubular necrosis, degeneration, desquamation, thickening of basement membrane and luminal cast formation;	[124]
	Antioxidant	Decreased the renal lipid peroxidation; Increased total sulfhydryl group, glutathione, vitamin C and vitamin E, and antioxidant enzymes, such as superoxide dismutase, catalase, glutathione peroxidase, glutathione-S-transferase, glutathione reductase, and glucose 6-phosphate dehydrogenase.	
Ethambutol hydrochloride-induced AKI	Renoprotective	Reduced the elevated levels of serum uric acid, BUN, and SCr;	[20]
	Antioxidant	Increased the superoxide dismutase activity.	
NaF-induced AKI	Antioxidant	Increased superoxide dismutase, glutathione, and catalase levels.	[121]
Methotrexate-induced AKI	Renoprotective	Reduced the renal tubular degeneration and dilation;	[118,119]
	Antioxidant	Decreased the number of apoptotic cells and caspase-3 expression; Decreased the malondialdehyde levels; Increased superoxide dismutase, glutathione peroxidase, and catalase levels; Lowered apoptotic index.	
Mercury-induced AKI	Renoprotective	Decreased the renal accumulation of Hg in the kidney; Reduced urinary excretion of protein-based biomarkers, including clusterin, KIM-1, NGAL, MCP-1, TIMP- 1, and VEGF; Protected against renal proximal tubular damage; Reduced apoptotic cell death in the kidney.	[122]
Valproic acid-induced AKI	Antioxidant	Decreased the lipid peroxidation and protein carbonyl; Reduced glutathione and nonprotein thiol levels.	[25]
Ciprofloxacin-induced AKI	Renoprotective	Reduced the tubular infiltration, dilatation, and atrophy as well as the Bowman’s space, congestion, hemorrhage, and necrosis;	[29]
	Antioxidant	Decreased the malondialdehyde levels; Increased the superoxide dismutase and catalase activities.	
Manganese-induced AKI	Renoprotective	Counteracted Mn-induced morphological glomerular damage; Decreased the expression of GRP78, CHOP, and caspase-3 proteins.	[123]
Cisplatin-induced AKI	Renoprotective	Maintenance of renal blood flow, BUN and SCr levels, and sodium fractional excretion; Decreased the NGAL and KIM-1 excretion;	[24,117,126]
	Antioxidant	Reduced the rise in MDA and protein carbonyl; Increased the GSH, vitamin C, vitamin E, total antioxidant capacity, and total glutathione levels in the kidney tissue; Induced the gene expression and activities of catalase, superoxide dismutase, glutathione reductase, and glutathione peroxidase enzymes in the kidney tissue;	
	Anti-inflammatory	Inhibited expression and secretion of IL-1β, IL-6, and TNF-α; Reduced the activity of Mincle/Syk/NF-κB signaling in vitro; Downregulated the protein levels of Mincle, phosphorylated Syk, and NF-κB in kidney macrophages; Inhibited M1, upregulated M2 macrophage activity; Reduced the early activation of stress kinases ERK, JNK and p38. Lowered NF-κB pathway and effector caspase activations.	
Contrast-induced AKI	Renoprotective	Inhibited the increase in SCr and albuminuria accompanied by a lower decrease in the GFR.	[120]
**Sepsis-induced AKI models**			
Lipopolysaccharide-induced AKI	Renoprotective	Relieved kidney dysfunction; Decreased the histopathological damage; Lowered the BUN and SCr levels. Reduced the inflammatory cell accumulation;	[24,130]
	Anti-inflammatory	Inhibited the Toll-like receptor-4, MyD88, and TRAF-6 expressions and NF-κBp65 activation in the kidneys; Inhibited the TNF-α, IL-1β, and IL-6 levels; Blockaded the CD38 expression of the macrophages possessing ADP-ribosyl cyclase and cyclic ADP-ribose hydrolase; Inhibited the LPS-induced macrophage M1 polarization accompanied by diminished NF-κB signaling activation.	

## Abbreviations

3CL(pro)	3c-Like Protease
ACLF	Acute-On-Chronic Liver Failure
ADPR-cyclase	ADP-Ribosyl Cyclase
AKI	Acute Kidney Injury
AKIN	Acute Kidney Network
Akt	Protein Kinase B
AMPK	AMP-Activated Protein Kinase
ASK1	Apoptosis Signal-Regulating Kinase 1
BMDMs	Bone Marrow-Derived Macrophages
BUN	Blood Urea Nitrogen
cADPR-hydrolase	Cyclic ADP-Ribose Hydrolase
CoVs	Coronaviruses
COVID-19	Coronavirus disease 2019
DHAV	Duck Hepatitis A Virus
GFR	Glomerular Filtration Rate
GSH-Px	Glutathione Peroxidase
HBV	Hepatitis B Virus
HCV	Hepatitis C Virus
HIV	Human Immunodeficiency Virus
HIVAN	Human Immunodeficiency Virus-Associated Nephropathy
I/R	Ischemia and reperfusion
ICU	Intensive Care Unit
IL	Interleukin
IL-18	Interleukin-18
iNOS	Nitric Oxide Synthase
IRF5	Interferon Regulatory Factor 5
JNK3	C-Jun N-Terminal Kinase 3
KDIGO	Kidney Disease: Improving Global Outcomes
KIM-1	Kidney Injury Molecule-1
L-FABP	Liver-Type Fatty Acid-Binding Protein
LPS	Lipopolysaccharide
MDA	Malondialdehyde
MERS-CoV	Middle East Respiratory Syndrome-Related Coronavirus
MMP-1	Matrix Metalloproteinase-1
MyD88	Myeloid Differentiation Protein
NF-κB	Nuclear Factor κB
NGAL	Neutrophil Gelatinase-Associated Lipocalin
PI3K	Phosphoinositide 3-Kinase
RBF	Renal Blood Flow
RIFLE	Risk, Injury, Failure, Loss, End-Stage Kidney Disease
RNS	Reactive Nitrogen Species
ROS	Reactive Oxygen Species
SARS	Severe Acute Respiratory Syndrome
SARS-CoV	Severe Acute Respiratory Syndrome Coronavirus
SCr	Serum Creatinine
SOD	Superoxide Dismutase
SYK	Tyrosine-Protein Kinase
TGF-β1	Transforming Growth Factor Beta
TNF-α	Tumor Necrosis Factor Alpha
TRAF6	Tumor Necrosis Factor Receptor-Associated Factor 6
